# MiR-205 Is Progressively Down-Regulated in Lymph Node Metastasis but Fails as a Prognostic Biomarker in High-Risk Prostate Cancer

**DOI:** 10.3390/ijms141121414

**Published:** 2013-10-29

**Authors:** Charis Kalogirou, Martin Spahn, Markus Krebs, Steven Joniau, Evelyne Lerut, Maximilian Burger, Claus-Jürgen Scholz, Susanne Kneitz, Hubertus Riedmiller, Burkhard Kneitz

**Affiliations:** 1 Department of Urology and Paediatric Urology, University Hospital Würzburg, Oberdürrbacher Str. 6, Würzburg 97080, Germany; E-Mails: martin.spahn@insel.ch (M.S.); krebs_m@klinik.uni-wuerzburg.de (M.K.); burger_m2@klinik.uni-wuerzburg.de (M.B.); Prof.Riedmiller@klinik.uni-wuerzburg.de (H.R.); 2 Department of Urology, University Hospital Bern, Holligen, Bern 3010, Switzerland; 3 Department of Urology, University Hospitals Leuven, Herestraat 49, Leuven 3000, Belgium; E-Mail: steven.joniau@uzleuven.be; 4 Department of Pathology, University Hospitals Leuven, Herestraat 49, Leuven 3000, Belgium; E-Mail: evelyne.lerut@uzleuven.be; 5 Microarray Core Unit, Interdisciplinary Center for Clinical Science, University of Würzburg, Versbacher Straße, Würzburg 97080, Germany; E-Mail: scholz_c@klinik.uni-wuerzburg.de; 6 Department of Physiological Chemistry, University of Würzburg, Am Hubland, Würzburg 97074, Germany; E-Mail: susanne.kneitz@uni-wuerzburg.de

**Keywords:** high-risk prostate cancer, microRNA, miR-205, prognosis, biomarker

## Abstract

The treatment of high-risk prostate cancer (HRPCa) is a tremendous challenge for uro-oncologists. The identification of predictive moleculobiological markers allowing risk assessment of lymph node metastasis and systemic progression is essential in establishing effective treatment. In the current study, we investigate the prognostic potential of miR-205 in HRPCa study and validation cohorts, setting defined clinical endpoints for both. We demonstrate miR-205 to be significantly down-regulated in over 70% of the HRPCa samples analysed and that reconstitution of miR-205 causes inhibition of proliferation and invasiveness in prostate cancer (PCa) cell lines. Additionally, miR-205 is increasingly down-regulated in lymph node metastases compared to the primary tumour indicating that miR-205 plays a role in migration of PCa cells from the original location into extraprostatic tissue. Nevertheless, down-regulation of miR-205 in primary PCa was not correlated to the synchronous presence of metastasis and failed to predict the outcome for HRPCa patients. Moreover, we found a tendency for miR-205 up-regulation to correlate with an adverse outcome of PCa patients suggesting a pivotal role of miR-205 in tumourigenesis. Overall, we showed that miR-205 is involved in the development and metastasis of PCa, but failed to work as a useful clinical biomarker in HRPCa. These findings might have implications for the use of miR-205 as a prognostic or therapeutic target in HRPCa.

## 1. Introduction

High-risk prostate cancer (HRPCa, defined by Gleason score ≥ 8 and/or pT ≥ 3 and/or PSA ≥ 20 ng/μL) is a PCa subgroup with varying risk of biochemical progression (BCR), clinical failure (CF) and cancer-specific mortality (CSM) following radical prostatectomy [1]. Today, uro-oncologists are faced with the complex decision about the type and timing of primary treatment [2]. While the key event of lymph node metastasis, which often remains undiscovered prior to initial treatment, impacts significantly upon management, reliable preoperative detection with current diagnostic imaging remains unsatisfactory [3].

Thus, new prognostic markers for HRPCa are necessary, especially if therapeutic targets are to be met. Numerous potential prognostic biomarkers have been identified but none of these has entered broader clinical use yet [4]. Among moleculobiological markers, the more recently described non-coding microRNAs (miRs) show promising results [5]. MiRs are small RNAs (19–25 nt long) regulating gene expression by binding to mRNA sequences and repressing target gene expression post-transcriptionally, either by inhibiting translation or by promoting RNA degradation [6]. Alterations of miR expression were detected in various cancer entities including PCa [7–14]. Functional studies have shown miRs to either function as tumour suppressors or oncogenes depending on the modulation of different target genes [8]. In addition, miR expression profiles stratify cancers by clones and level of differentiation suggesting that miRs are involved in cancer progression [9,10].

However, understanding the regulation and function of miRs in normal and neoplastic tissues remains a great challenge. Recent studies of several miRs exhibited both positive and negative correlation between expression and tumour type implicating a very complex and dynamic function of miRs for tumour development and progression [8–10]. Also, miR-205 either acts as a tumour suppressor or as an oncogene depending on the regulation of potential target genes in the context of the specific tumour [15,16] It has been shown that miR-205 is frequently down-regulated in a variety of cancer entities and its tumour-suppressive action is well studied [17–27]. MiR-205 impacts crucial pathways involved in proliferation, invasion and angiogenesis by mediating the expression of multiple oncogenes like *HER3*, *VEGF-A*, or *MED1* [20,21,27]. Further important pathways in cancerogenesis regulated by miR-205 are NFKβ-enhancing, epithelial to mesenchymal transition (EMT) and induction of chemotherapy resistance [23–27]. In contrast to this tumour-suppressive function of miR-205, several studies have described miR-205 as up-regulated in lung cancer or HNSCC (head and neck cell squamous carcinoma) suggesting an oncogenic role [28,29]. The oncogenic function of miR-205 is substantiated by suppression of potential tumour-suppressor genes like *PTEN*, *TRAF2* or *SHIP2* and by promotion of aggressive lung and squamous carcinomas [28–31].

With regard to PCa, miR-205 has been reported to be frequently down-regulated and it was suggested that down-regulation might be associated with a poorer prognosis in PCa [17–19,27]. Nevertheless, the impact of miR-205 as a potential prognostic outcome marker in PCa has not previously been investigated in a large complementary PCa collective until now. The HRPCa cohorts used in this study are a prerequisite for the validation and optimization of miR-based prediction of lymph node metastasis and cancer progression in PCa due to the relatively high rates of relevant events in this subgroup in comparison with low- or intermediate-risk series [10,32]. The primary aim of this study is to assess the validity of a miR-205-based prognostic tool using two well-characterized HRPCa cohorts.

## 2. Results

### 2.1. MiR-205 Is Under-Expressed and Its Reconstitution Leads to Proliferation—Inhibition in PCa Cells

**Figure 1:**
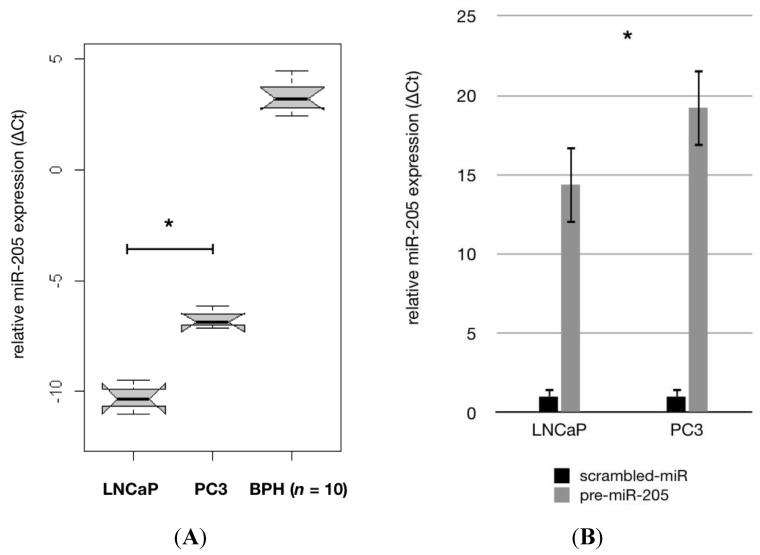

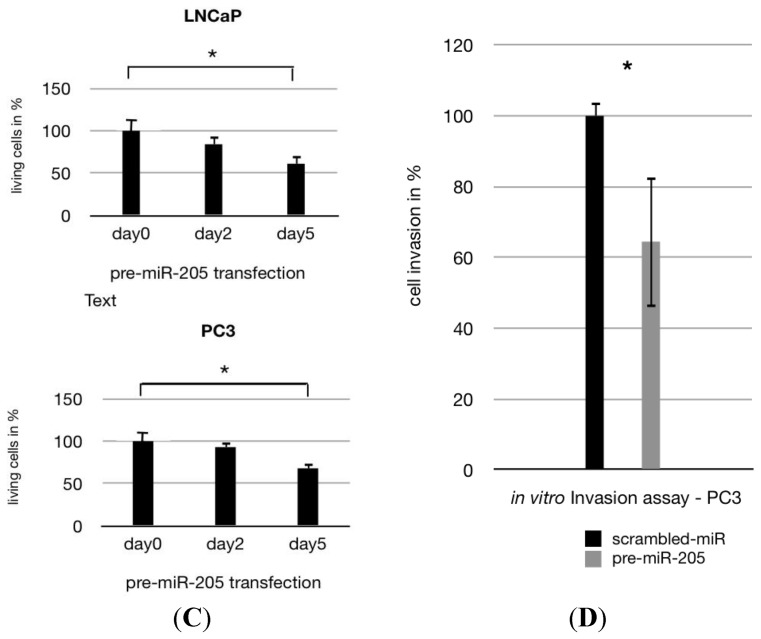
(**A**) Boxplots of Real-Time polymerase chain reaction (PCR) assays in PCa cell lines LNCaP and PC-3 compared to benign hyperplastic prostate tissue (BPH, *n* = 10); (**B**) Histograms indicating transfection efficiency of transient miR-205. Transfection was verified by measuring miR-205 expression via Real-Time Quantitative PCR (qRT-PCR) in pre-miR-205 transfected cells compared to cells transfected with scrambled miRNA. Error bars represent the standard deviation of three independent experiments; (**C**) MTS proliferation assays in precursor-miR 205 transfected cell lines LNCaP and PC3. Histograms indicate the percentage of living cells compared with cells transfected with scrambled miRNA after transient transfection with pre-miR-205 (day 0) at given intervals (2 and 5 days); (**D**) MiR-205 up-regulation reduces cell migration of PCa cells. PC-3 cells were transfected with pre-miR-205 or scrambled miRNA. Migration of PC-3 cells was measured over 6 h in a Transwell^®^ cell culture chamber. Four chambers from three different experiments were analysed (*p* = 0.005). Error bars represent the standard deviation of three independent experiments. * indicates *p* < 0.01. *p*-values were calculated in cell lines and BPH tissue (**A**) using one-way ANOVA with *post hoc* testing (Tukey’s test). Student’s unpaired *t*-test was used for transfection efficiency and proliferation/invasion experiments (**B**–**D**).

We analysed the expression of miR-205 in LNCaP and PC-3 cells via qRT-PCR and demonstrated that androgen-sensitive LNCaP cells showed a lower miR-205 expression than androgen-insensitive PC-3 cells (Figure 1A, *p* < 0.05). Transfection efficiency of the cell lines was verified using qRT-PCR (Figure 1B, *p* < 0.01). Proliferation assays in the cell lines showed that miR-205 transfection reduced cell proliferation significantly (Figure 1C, *p* ≤ 0.01). On day five *post* transfection 61% and 68% of LNCaP and PC-3 cells were viable. We then analysed whether the expression of miR-205 showed an impact on the invasive activities of the cells. Boyden chamber invasion assays revealed reduced invasion levels in PC-3 cells transfected with pre-miR-205 (Figure 1D). These results demonstrate that miR-205 acts as a tumour suppressor in PCa cells by regulating cell growth and invasiveness.

### 2.2. MiR-205 Is Under-Expressed in a HRPCa and Shows Increasing Down-Regulation in Lymph Node Metastases

To prove whether miR-205 is down-regulated in high-risk PCa cases, we subsequently analysed miR-205-expression in a HRPCa study collective (*n* = 105) and in BHP samples (*n* = 10) serving as controls. Clinical demographics of the study cohort and the BHPs are summarized in Table 1 and respectively in Table S1. In our study group mean miR-205 expression was significantly lower in the cancerous samples than in BPH controls. 98.1% of the tumour samples showed a lower miR-205 expression compared with mean expression of BPH samples (*p* ≤ 0.01, comparing absolute Δ*C*t levels of individual samples to the mean Δ*C*t expression of 10 BPH samples). Using the ΔΔ*C*t method we found that 82 of 105 cancer samples (78.1%) had a more than two-fold reduction in miR-205 expression if compared to the control tissue (*p* ≤ 0.01, see Figure S1). From these results we concluded that miR-205 is frequently and strongly reduced in HRPCa cases.

**Figure 2:**
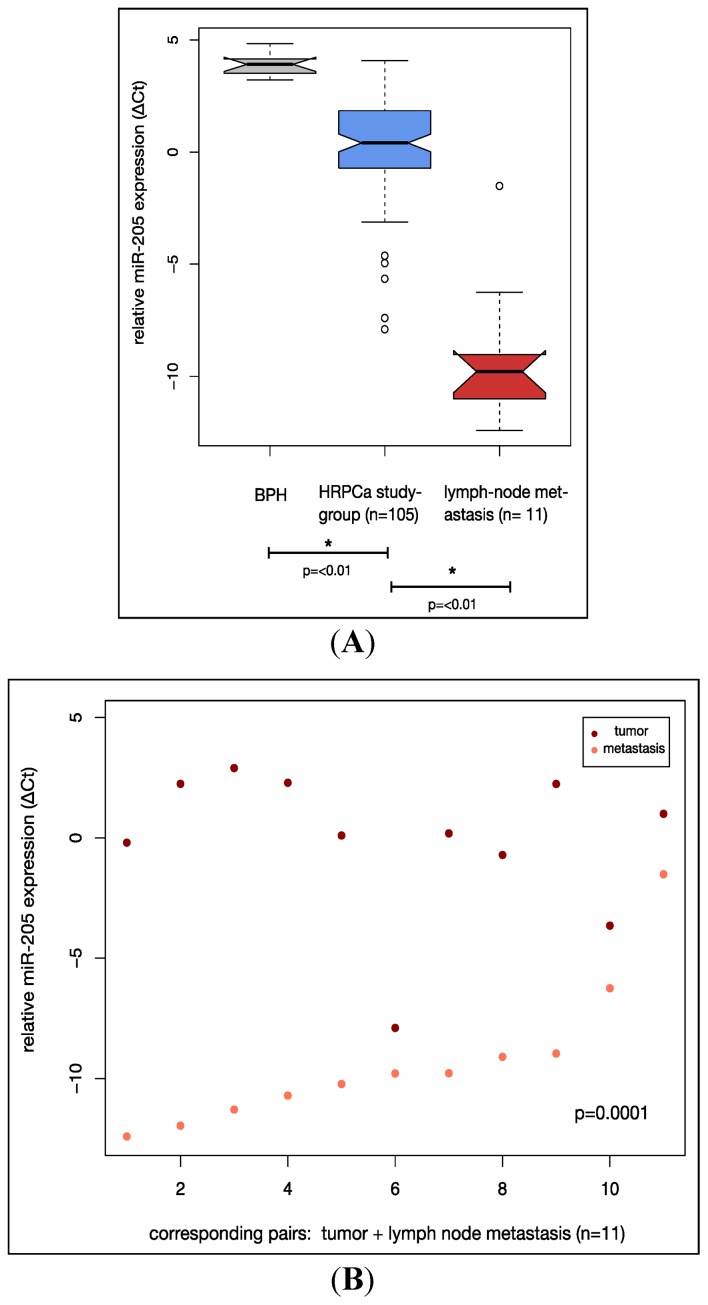
Increasing down-regulation of miR-205 expression in a HRPCa cohort (*n* = 105) and lymph node metastases. Relative miR-205 expression was analysed by the Δ*C*t method using qRT-PCR in all samples. (**A**) MiR-205 expression was analysed in 10 BPH tissues (**left box**), 105 HRPCa specimen (**middle box**) and 11 lymph node metastases (**right box**). Significant reductions in expression levels are marked by * (*p* <0.01). *p*-values were calculated using one-way ANOVA with *post hoc* testing (Tukey’s test); (**B**) Dot plot of miR-205 expression in synchronous lymph node metastasis. 11 pairs of primary tumour samples and corresponding lymph node metastases were analysed. *p*-value was calculated by Student’s paired *t*-test.

Next, we analysed the expression of miR-205 in lymph node metastasis. As shown in Figure 2A the expression of miR-205 in lymph node metastases (*n* = 11) was significantly and increasingly reduced when compared with the mean miR-205 expression in primary PCa samples of the study cohort (*n* = 105) and BPH controls (*n* = 10). Figure 2B shows miR-205 expression in primary tumours and corresponding synchronous lymph node metastases (*n* = 11). We found miR-205 to be down-regulated in all analysed pairs (*p* = 0.0001) and observed a more than two-fold down-regulation in 8 of the 11 pairs (72.7%) indicating that lymph node metastases are characterized by a strong and increasing reduction of miR-205 expression when compared with the primary tumour.

### 2.3. MiR-205 Expression Is Not Significantly Linked to Clinical Prognostic Parameters in HRPCa

Based on the result that miR-205 is down-regulated in lymph node metastasis we suggested that progressive miR-205 down-regulation might be associated with clinical parameters in HRPCa. To correlate miR-205 down-regulation with advanced PCa stages, we related the clinical parameters of the HRPCa patients to corresponding miR-205 expression patterns.

**Figure 3:**
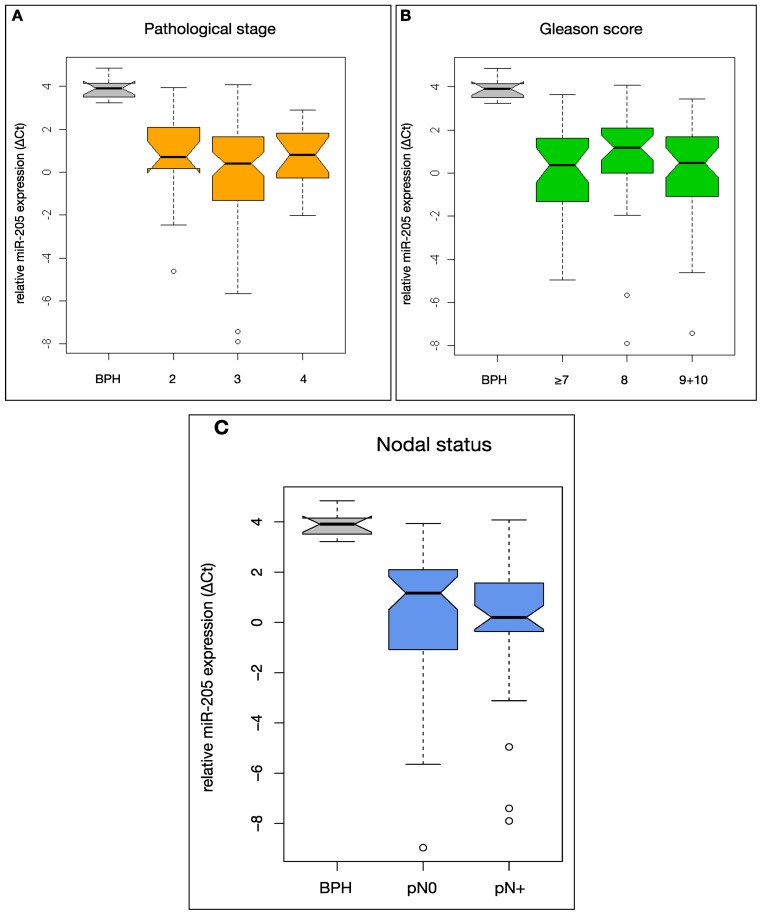

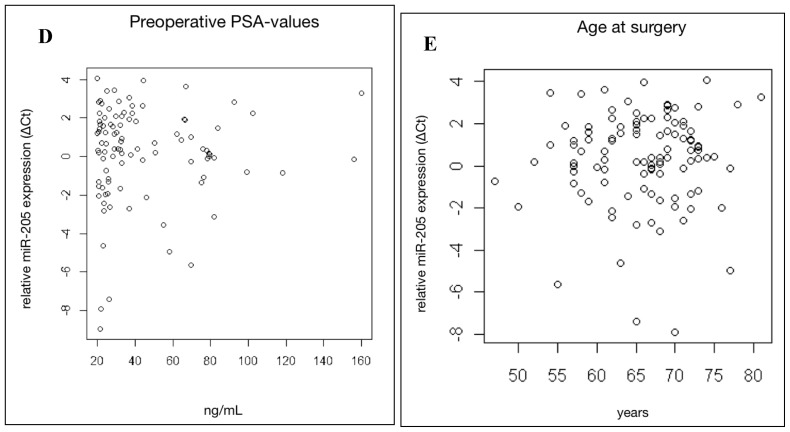
MiR-205 expression as related to different clinical parameters in the study cohort (*n* = 105). Relative miR-205 expression was analysed in all samples by the Δ*C*t method using qRT-PCR. (**A**), (**B**) and (**C**) Boxplots showing the relation of miR-205 expression with the pathological tumour stage, Gleason score and the nodal status of the primary tumour in the study cohort in comparison to that in 10 BPH tissues. *p*-values were calculated using one-way ANOVA (**A** and **B**) and Student’s unpaired *t*-test (**C**). (**D**) and (**E**) Dot plots showing the association of miR-205 expression with preoperative PSA-values and age at surgery.

Figure 3 shows miR-205 expression in HRPCa cases separated by the respective clinical parameters in comparison with BPH. All clinical parameters show a significantly lower miR-205 expression in every stage compared to BPH tissue as control (*p* < 0.01). The pathological stage (*p* = 0.793), Gleason score (*p* = 0.956) and the nodal status (*p* = 0.31) of the primary tumour at surgery or high/low preoperative parameters (PSA-values, age at surgery) could not be stratified by miR-205 expression.

### 2.4. MiR-205 as a Prognostic Factor in HRPCa

**Figure 4:**
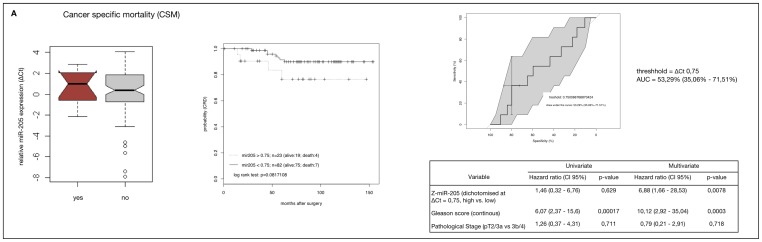

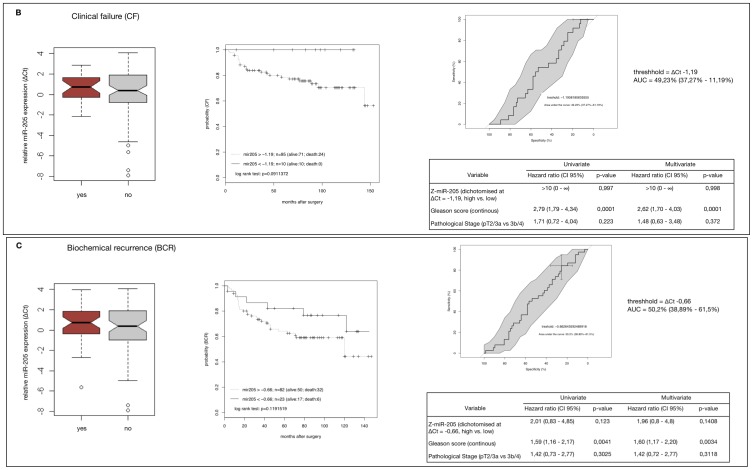
MiR-205 as a prognostic marker in a HRPCa study cohort (*n* = 105) for the clinical endpoints (**A**) Cancer-specific mortality (CSM); (**B**) Clinical failure (CF) and (**C**) Biochemical recurrence (BCR). **Left plots**: boxplots showing relative miR-205 expression (Δ*C*t sample) in patients with and without the indicated endpoints. **Middle plots**: Kaplan–Meier estimates in HRPCa patients with and without the respective endpoints. Patients were dichotomised by miR-205 expression according to optimal cut-off values for each endpoint calculated by ROC (**upper right plots**). *p*-values were calculated using log-rank test. Lower right tables: uni- and multivariate Cox-regression analysis regarding dichotomised miR-205 expression, Gleason score and combined pT score (pT2c/3a *vs*. pT3b/4) for the time to the respective end-points. *p*-values were calculated using Wald test.

To evaluate if miR-205 down-regulation might be prognostic in HRPCa we performed Kaplan–Meier estimates and Cox proportional hazard models. To stratify cut-off values for the optimal trade-off between specificity and sensitivity, *Z*-score normalised values of relative miR-205 expression in the specimen of the study group were dichotomised for CSM, BCR and CF using receiver operating characteristic (ROC) curves. AUC-values of the ROC curves ranged between 49.2% and 53.3%. (upper right plots, Figure 4A–C), indicating no discriminative value and very limited predictive power. Despite this, no significant difference could be found in miR-205 expression between the subgroups for all endpoints analysed (left plots, Figure 4A–C) and no correlation could be demonstrated between miR-205 down-regulation and CSM, CF or BCR using Cox regression analysis. Also, undichotomised values gave no significant results either (data not shown). Paradoxically however, in Kaplan–Meier estimates, up-regulation of miR-205 expression was associated with a worse prognostic outcome, but this trend only reached marginal significance for CSM and CF (middle plots, Figure 4A–C, CSM, *p* = 0.08; CF, *p* = 0.09; BCR, *p* = 0.12).

### 2.5. Down-Regulation of MiR-205 Fails as a Prognostic Factor in HRPCa

To confirm the findings observed in the study cohort (*n* = 105), we analysed a complementary HRPCa-cohort (*n* = 78) after adjusting the mean of each sample’s miR-205 expression values to 0 and standard deviation to 1 by *Z*-score normalisation, thus improving comparability between cohorts. Clinical demographics of the validation cohort are summarized in Table 1. *Z*-scores of the validation cohort were dichotomised using the same thresholds as for the first cohort.

**Figure 5:**
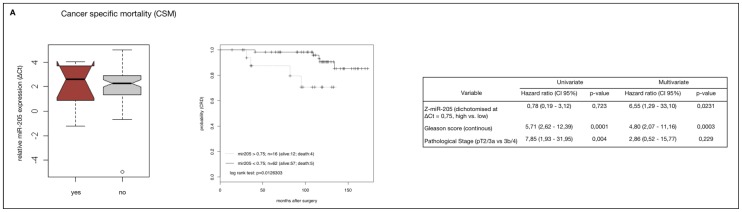

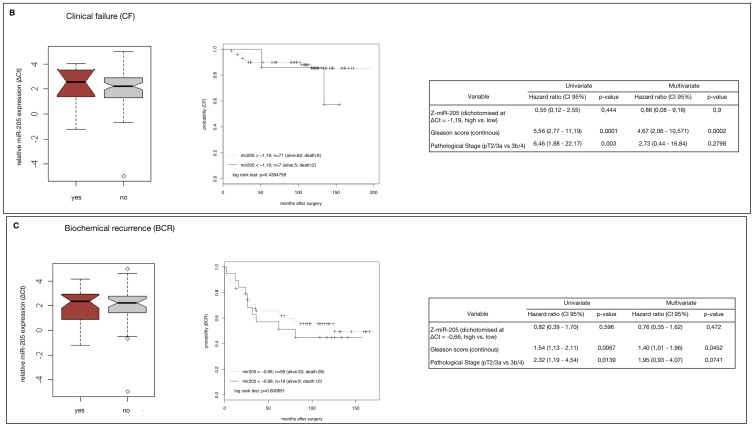
MiR-205 as a prognostic marker in a HRPCa validation cohort (*n* = 78) for clinical endpoints (**A**) cancer-specific mortality (CSM); (**B**) clinical failure (CF) and (**C**) biochemical recurrence (BCR). **Left plots**: boxplots showing relative miR-205 expression (Δ*C*t sample) in patients with and without the indicated endpoints. **Middle plots**: Kaplan–Meier estimates in HRPCa patients with and without the respective endpoints. Patients were dichotomised by miR-205 expression according to optimal cut-off values for each endpoint calculated by ROC (**upper right plots**). *p*-values were calculated using log-rank test. Lower right tables: uni- and multivariate Cox-regression analysis regarding dichotomised miR-205 expression, Gleason score and combined pT score (pT2c/3a *vs*. pT3b/4) for the time to the respective end-points. *p*-values were calculated using Wald test.

We also found a significant down-regulation of miR-205 in the validation cohort (Figure S2). 78.2% (61 of 78 samples) of the tumour samples of the validation cohort were characterised by a more than two-fold reduction of miR-205 expression compared to mean expression in 10 BPH samples (*p* ≤ 0.01; Figure S1). As already shown in the study cohort we found no association of miR-205 down-regulation with clinical parameters (*p* > 0.05, see Figure S3) or correlation of miR-205 expression to CSM, CF or BCR (Figure 5) using univariate Cox regression analysis.

However, we confirmed the finding that miR-205 up-regulation (Δ*C*t > 0.75) was associated to reduced CSM in Kaplan–Meier analysis (*p* = 0.02). Multivariate analysis also confirmed that miR-205 up-regulation was an independent predictor of CSM (*p* = 0.0003), but not for CF or BCR in the validation cohort.

## 3. Discussion

Emerging evidence shows that miRs not only impact upon physiologic development such as embryogenesis but also upon cancerogenesis, including PCa development [7]. Whilst numerous reports dwell on specific aberrant miR expression profiles, only limited information is available on the potential use of miRs as biomarkers and clinical prognosticators in PCa. Therefore, we analysed the expression of miR-205, previously reported to be dysregulated in cancer, in two well-characterized HRPCa series and in *in vitro* cell culture models.

We found miR-205 to be significantly and frequently under-expressed in both analysed HRPCa cohorts and in PCa cell lines, indicating that down-regulation of miR-205 is a very common process in HRPCa. These findings are consistent with previous studies on miR-205 expression describing miR-205 as down-regulated in various types of cancer like prosate cancer, breast cancer or Barrett’s oesophagus [11–14,20–22]. Using PCa cell lines we demonstrated a tumour-suppressive function of miR-205 affecting cellular growth, adhesion and migration and confirmed earlier results that describe miR-205 as a tumour suppressor [17–19,23,27]. Moreover, three recent studies suggested that miR-205 down-regulation might be associated with an adverse outcome in PCa patients [17,26,27]. The study by Hulf *et al*. showed that epigenetic-induced repression of miR-205 is associated with poorer prognosis in localized PCa cases. Tucci *et al*. demonstrated that p63 regulates the expression of miR-205 in PCa and revealed that defects in the p63/miR-205 axis correlate to a poorer outcome in PCa. Both studies were limited by the usage of biochemical recurrence as the main clinical endpoint, which has a lower prognostic relevance than clinical recurrence or cancer-specific mortality of the patients. Using tumour samples isolated from a TURP (transurethral resection of the prostate) cohort recruited in the pre-PSA era, the third study by Hagman *et al*. indicates that miR-205 down-regulation is associated with adverse outcome in PCa patients, thus potentially functioning as a prognostic marker in PCa. Nevertheless, none of these recent studies correlated miR-205 expression with cancer-specific mortality or clinical recurrence using a high-risk PCa cohort. Since the prognostic power of miR-205 expression has not been previously determined in an untreated HRPCa cohort, we correlated miR-205 expression to firm clinical endpoints using two well characterized HRPCa series serving as independent study and validation cohorts [10–32]. Contradictory to the observed association of biochemical relapse with loss of p63/miR-205 function or with epigenetic-induced repression of miR-205, we showed that strong down-regulation of miR-205 was not associated with CSM, CF, BCR or clinical parameters in HRPCa patients. This result accords with miRNA expression studies showing that miR-205 does not correlate to outcome in PCa [11–14]. Even if our study is limited to the use of high-risk samples only, our analysis strongly implicates that down-regulation of miR-205 in primary PCa cases fails to function as an independent prognostic marker in PCa. Based on these results it will be of interest to analyse the potential of epigenetic-induced or p63-dependent down-regulation of miR-205 as a prognostic marker in HRPCa.

In contrast to the expected progressive down-regulation of miR-205 in aggressive PCa cases, we even observed a moderate up-regulation of miR-205 in patients with higher risk for CSM and were able to confirm this result in a validation cohort suggesting potentially different roles of miR-205 in various PCa risk-groups. This paradoxical observation was backed by the finding that miR-205 expression is significantly lower in the indolent, androgen-sensitive cell line LNCaP compared with the aggressive PC-3 cell line. This antithetic regulation of miR-205 in more aggressive PCa samples may seem paradoxical, but might be consistent with the observation that miR-mediated regulation of target molecules may be determined by the specific microcellular environment and dynamic regulation [33,34].

Such an environmental determination for miR-205 seems perfectly possible, since several studies describe miR-205 as up-regulated in several cancer types like lung cancer or HNSCC (head and neck cell squamous carcinoma) [28,29]. An oncogenic role for miR-205 is supported by inhibition of tumour suppressors like *PTEN*, a gene frequently mutated in PCa, or *SHIP2* [28–31]. Thus, miR-205 might act as a tumour suppressor or an oncogene in different tissues and at different stages of tumour progression [11]. Such a pivotal role for miR-205 has been suggested in a recent review by Greene *et al.* looking at potential target genes of miR-205 [16]. Nevertheless, the relevance of the observed correlation between miR-205 up-regulation and a poorer outcome in PCa patients has to be questioned since the AUC values we found by ROC computation showed an average of around 50%. This result weakens the assumption that miR-205 up-regulation could predict CSM in either cohort reliably. We are planning to carry out further expression studies using enlarged high-risk patient cohorts to clarify the role of miR-205 up-regulation as predictor for CMS in various risk-groups of PCa.

Several independent studies have shown that miR-205 was markedly down-regulated in cells that undergo ephitelial to mensynchymal transition (EMT) by targeting *ZEB1*, *SIP1* or *PKCe* [23–27]. EMT is considered an essential process in tumour metastasis, suggesting that miR-205 down-regulation might be associated to metastasis in PCa. In this context it was recently reported that miR-205 down-regulation is directly involved in the development of androgen independency in PCa cells and is correlated to the occurrence of metastases [17,19]. The last finding by Hagman *et al*. was demonstrated in transurethral-acquired localized tumour samples of castration-resistant PCa (CRPC) patients, who had received neoadjuvant hormonal or radiation therapy [17]. In contrast, we could not find progressive miR-205 down-regulation in primary cancers of patients with lymph node metastasis compared to patients with localised disease. However, all samples used in the present study were taken from patients with hormone naïve disease, who never received adjuvant therapy. Our finding that miR-205 expression in primary PCa cases is not associated with the occurrence of metastasis is confirmed by studies screening for metastasis-associated miRs. These studies showed no differences in miR-205 abundance between organ-confirmed tumours and those with extraprostatic disease extension [10,11,14]. Nevertheless, we observed strong and increasing down-regulation of miR-205 in a set of lymph node metastases if compared to the expression in corresponding primary tumour samples. Depending on these results we concluded that down-regulation of miR-205 is critically involved in invasion, adhesion and homing of PCa cells after migration from the original location of the prostate to the exptraprostatic tissue. This notion is supported by our *in vitro* results showing that miR-205 regulates migration and adhesion of PCa cells independently from the proliferative activity of the cells. One explanation for the differential expression of miR-205 between the primary PCa and its lymph node metastasis might be the observed pivotal role of miR-205 as an ongogenic or tumour-suppressive miR. It might be critical for PCa cells to balance down-regulation of miR-205 expression in the microenvironment of normal prostate tissue avoiding severe up-regulation of potential tumor-suppressive miR-205 target genes like *PTEN* or *SHIP2*. In contrast, metastatic cells undergoing EMT increase their cell mobility to migrate from the original location and to home to lymph nodes or other extraprostatic tissues. This process might be associated with strong and progressive down-regulation of miR-205 increasing the expression of oncogenes involved in EMT, like *ZEB1* or *SIP1* [14,23–26]. To prove this hypothesis, we are planning to elucidate the correlation of miR-205 to relevant target genes in primary PCa cases and its metastasis in the future.

Overall, there is clear evidence that miR-205 acts as a tumour suppressor in PCa, interfering with processes involved in castration resistance and EMT [17,19,25]. Our present data promote a tumour-suppressive role of miR-205 in HRPCa due to its frequent down-regulation in tumourous tissue. Moreover, lymph node metastasis progressively down-regulate miR-205 in comparison to the primary tumour suggesting that miR-205 is critically involved in distinct processes leading to migration, invasion or homing of metastatic PCa cells. However, down-regulation of miR-205 clearly failed to act as a useful clinical predictor for progression or metastasis in HRPCa. Paradoxically, we found a tendency that miR-205 up-regulation correlates with adverse outcome of prostate cancer patients. Depending on pivotal functions of miR-205 in cancerogenesis we suggest that miR-205 expression might be tightly controlled at different tumour stages affecting the expression of either tumour suppressors or oncogenes.

## 4. Experimental Section

### 4.1. Cell Lines

LNCaP (androgen-sensitive) and PC-3 (androgen-independent) human prostate carcinoma cell lines were obtained from ATCC (American Type Culture Collection, Chicago, IL, USA) and cultured in RPMI 1640 (PAA, Pasching, Austria) supplemented with 10% fetal calf serum (FCS), 100 U/mL penicillin, 100 mg/L streptomycin and 2 mmol/L glutamine.

### 4.2. Cell Transfection

PC-3 and LNCaP cells were plated in a final concentration of 3 × 10^4^ to 5 × 10^4^ per well. After 24 h, OPTI-MEM medium mixed with Lipofectamine 2000 and precursor-miR-205 or scrambled miRNA as control was applied to selected wells following the manufacturer’s protocol.

### 4.3. Cell Proliferation Assay

PC-3 and LNCaP cells were plated at 3 × 10^4^ to 5 × 10^4^ per well in triplicate in 96-well plates. MiR-205 transfection was carried out after 24 h using the method described above. After 2 and 5 days, cells were analysed with MTS CellTiter96 Proliferation Assay (Promega, Madison, WI, USA, at 490 nm with a monochromator (Biorad, Hercules, FL, USA) using the manufacturer’s protocol.

### 4.4. *In Vitro* Invasion Assay

A modified Boyden chamber assay was performed as described by Grunewald *et al*. [35]. PC-3 cells were cultivated and transfected with pre-miR-205 or scrambled miRNA as described in 4.1. After 48 h incubation and overnight starvation in DMEM plus 0.5% FCS, cells were seeded in the upper chamber of BSA-coated 8 μM pore size Transwell^®^ Boyden chambers (Corning star, Cambridge, MA, USA). 10% FCS growth medium was added to the bottom chamber as an attractant and cells were allowed to migrate through the membrane for 6 h. After removal of all cells remaining at the upper surface using a cotton carrier the lower surfaces of the membranes were stained for 30 s in a solution of 1% (*w*/*v*) crystal violet. Membranes were then washed with distilled water. Cell-associated crystal violet was extracted in 10% acetic acid and measured at 595 nm absorbance. The experiments were performed in triplicate.

### 4.5. Patients and Samples

Men with HRPCa who had undergone radical retropubic prostatectomy (RRP) between 1987 and 2005 at the Community Hospital of Karlsruhe, Germany and the Department of Urology and Pediatric Urology of the University Medical Center Würzburg, Germany (study cohort characterized previously by Spahn *et al*. [10]) and the University Hospital Leuven, Belgium (validation cohort) were identified in the European Clinical and Translational High-Risk Prostate Cancer Research Group database (EMPaCT) and included into this study. Our study cohort also included 11 patients of which we had access to samples of synchronous lymph node metastases. All RRP and lymph node metastasis samples were paraffin-embedded and areas with >90% cancerous tissue were selected; likewise prostate adenomectomy samples from the Department of Urology and Pediatric Urology of the University Medical Center, Würzburg were identified in a respective database and areas with >80% adenoid tissue were selected (for clinical demographics see Table S1). PCa specimens (whole mount sections, 4 mm intervals) were staged and graded according to the 2002 TNM classification and the Gleason grading system by two experienced uro-pathologists. All patients were pre-operatively subjected to digital rectal examination, transrectal ultrasound, assessment of PSA, CT and bone scan. None of the patients had received neoadjuvant hormonal-, radiation- or chemotherapy. Follow-up visits including digital rectal examination, transrectal ultrasound and assessment of PSA were scheduled every 3 months for the first 2 years after RRP, every 6 months in the following 3 years, and annually thereafter. BCR was defined as PSA ≥ 0.2 ng/mL on two consecutive follow-up visits. CF was defined either as histologically proven local recurrence or distant metastasis confirmed by CT or bone-scan. Cause of death was verified by chart review and CSM was defined as death due to prostate cancer. Overall survival (OS) was defined as time from RRP to death from any cause, cancer-specific survival (CSS) as the time from RRP to death attributed to PCa or complications of the disease. Clinical and pathological characteristics, BCR- and CF-free survival, CSM and OS were comparable for both cohorts (see Table 1 and Figure S4). After a median follow-up of 78 months (1–154) for the study cohort and 117 months (14–173) for the validation cohort a total of 24 men (22.9%) and 11 men (13.9%) suffered CF and 11 (10.5%) and 9 (11.4%) CSM, respectively. The estimated 10-year overall and cancer-specific survival rates were comparable for both patient groups (61.3% and 87.3% for the study cohort and 71.1% and 81.5% for the validation cohort, see Figure S4).

### 4.6. qRT-PCR

Total RNA was extracted from PCa samples and BPH tissues with Total RNA Extraction Kit (Life Technoligies, Carlsbad, NM, USA). For cell lines, TRIzol Agent (Life Technoligies, Carlsbad, NM, USA) was applied 48 h after transfection and RNA was extracted and precipitated using Chloroform and Isopropanol. The RNA concentration was determined with a Bioanalyser (BIORAD, Hercules, FL, USA). cDNA was synthesized according to the TaqMan miR Assay protocol (Life Technoligies, Carlsbad, NM, USA). Mature miR expression was quantified in tissue samples with TaqManR miR assay kits and an Applied Biosystems 7900 HT system. We followed the protocol provided in the manufacture’s instructions (Applied Biosystems, Foster City, CA, USA). The expression of RNU6b was used for normalization. Relative miR expression was calculated with the comparative Δ*C*t-method (Δ*C*t sample = *C*t sample – *C*t RNU6b; Δ*C*t BPH = *C*t BPH – *C*t RNU6b). Fold changes were calculated using the ΔΔ*C*t-method (ΔΔ*C*t = fold change = 2^(Δ^*^C^*^t Sample – Δ^*^C^*^t BPH)^), whereas Δ*C*t BPH was determined as the mean of the Δ*C*t values of the 10 BPH samples. Calculations were carried out assuming equal RNA-concentrations and complete efficiancy of qRT-PCR.

### 4.7. Statistical and Bioinformatical Analysis

Statistical computation was achieved by using the statistical language R. Mean values of normally distributed data were compared with a two-sided unpaired student’s *t* test. More than two group means were differentiated by analysis of variance (ANOVA) with *post hoc* testing (Tukey’s test) if significant differences occurred. Various risk factors were correlated in uni- and multivariate Cox proportional hazard models to predict CSM, CF and BCR using the bioconductor package “survival.” Kaplan–Meier estimates were created using *Z*-score normalised and subsequently dichotomised data. For miR-205, optimal thresholds were calculated for dichotomisation by using the package “pROC.” (http://cran.r-project.org/web/packages/pROC/index.html). Significant associations were set as *p* < 0.01 (*).

## 5. Conclusions

We present our results of miR-205 expression in two of the largest HRPCa series reported to date serving as independent study and validation cohorts. Our present data promote a tumour-suppressive role of miR-205 in HRPCa due to its frequent and increasing down-regulation in tumourous tissue and in lymph node metastasis suggesting miR-205 to be critically involved in migration, invasion or homing of PCa cells. However, down-regulation of miR-205 clearly failed to act as a useful clinical predictor for progression or metastasis in HRPCa. Moreover, we observed a tendency that miR-205 up-regulation correlates with an adverse outcome for prostate cancer patients. These findings might have implications for the use of miR-205 as a prognostic marker and therapeutic target in PCa.

## Supplementary Information



## Figures and Tables

**Table 1:** Preoperative and postoperative patients’ characteristics of the HRPCa study cohort (*n* = 105) and the HRPCa validation cohort (*n* = 78).

	Study cohort	Validation cohort
Number of patients	105	78
Median Δ*C*t miR-205 expression (range)	0.33 (−7.9–4.1)	1.96 (−4.95–5.0)
Median follow-up, months (range)	78.3 (1–154)	117 (14–173)
Median Age, years (range)	65.9 (47–81)	63 (41–75)
Median PSA, ng/μL (range)	32 (20–160)	14 (2.7–95.3)

**Gleason Score (GS):**		

GS ≤ 6	2 (1.9%)	24 (30.4%)
GS =7	31 (29.5%)	37 (46.8%)
GS 8–10	72 (68.6%)	18 (22.8%)

**Pathological Stage (pT):**		

pT 2	17 (17.2%)	24 (34.2%)
pT3a	28 (26.7%)	35 (44.3%)
pT3b	44 (41.9%)	16 (24.1%)
pT4	16 (15.2%)	4 (5.1%)

**Nodal Status (LN):**		

LN neg	64 (61.0%)	70 (89.7%)
LN pos	41 (39.0%)	8 (10.3%)

**Relapse Events:**		

Biochemical recurrence	38 (36.2%)	36 (49.4%)
Clinical failure	24 (22.9%)	11 (13.9%)
Cancer-specific mortality	11 (10.5%)	9 (11.4%)
Any cause death	21 (20.0%)	16 (24.1%)
