# Supplementary material for: MiR-205 Is Progressively Down-Regulated in Lymph Node Metastasis but Fails as a Prognostic Biomarker in High-Risk Prostate Cancer
Source: Int J Mol Sci. 2013 Oct 29;14(11):21414–34. doi: 10.3390/ijms141121414 (PMC3856012; doi:10.3390/ijms141121414)
Supplement: Supplementary file 1 [file ijms-14-21414-s001.pdf]

Supplementary Information

Table S1. Demographics of BPH patients serving as controls.

	**BPH samples**
Number of samples	10
Age, years (mean)	67–81 (71.2)
PSA, ng/µL (mean)	2.1–8.0 (3.6)
Method of fixation	paraffin-embedded
Sample acquisition	
Adenomectomy	10
TURP	0
other	0

Figure S1. Visualisation of fold change in miR-205 expression in (**A**) a HRPCa study (n = 105) and (**B**) a HRPCa validation cohort (n = 78) compared to BPH tissue as control (n = 10). Fold changes were calculated using the $\Delta\Delta C$t-method as described in chapter 4.6. Fold changes are visualised in descending order in bar plots assuming equal RNA-concentrations and complete efficiency of qRT-PCR. Cut-offs indicating the samples which are characterised by significant reduction of miR-205 expression (more than two-fold reduction) are displayed in both plots (**black bars and arrows**). p-values were calculated using Student's unpaired t-test.

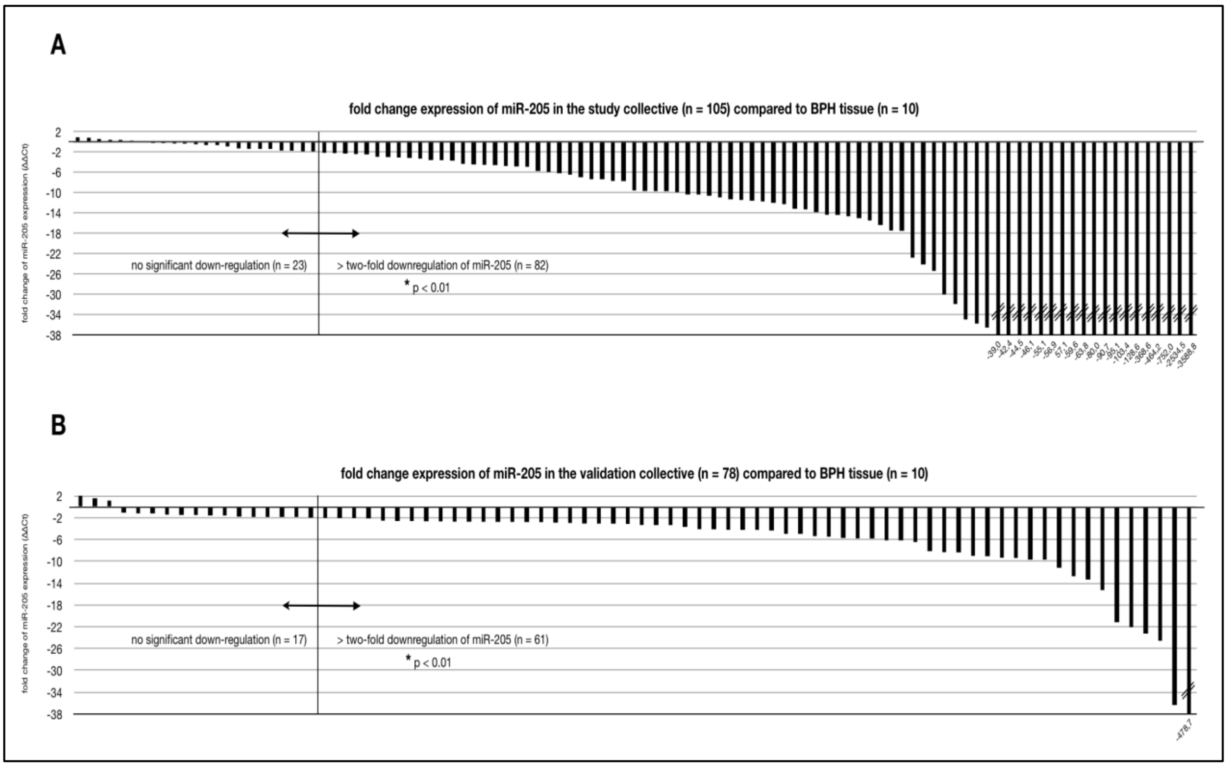

Figure S2. MiR-205 expression in a HRPCa validation cohort (n = 78). Relative miR-205 expression was analysed by the ΔCt method using qRT-PCR in all samples. miR-205 expression was analysed in 10 BPH tissues (**left plot**) and 78 HRPCa specimen (**right plot**). Significant reductions in expression levels are marked by * (p < 0.01). p-values were calculated by Student's unpaired t-test.

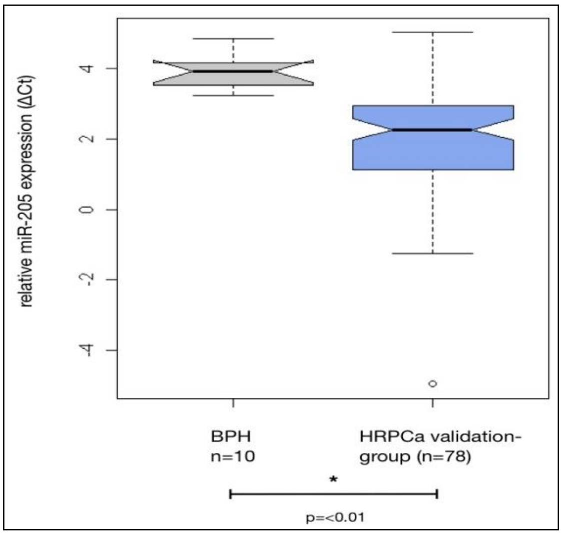

Figure S3. miR-205 expression in association with different clinical parameters in the validation cohort (n = 78). Relative miR-205 expression was analysed in all samples by the ΔCt method using qRT-PCR. (**A**), (**B**) and (**C**) boxplots showing the association of miR-205 expression with the pathological tumour stage, the Gleason score and the Nodal status of the primary tumour in the validation cohort in comparison to that in 10 BPH tissues. Black bars represent median expression of miR-205. P-values were calculated using one-way ANOVA (**A** and **B**) and Student's unpaired t-test (**C**). (**D**) and (**E**) dot plots showing the association of miR-205 expression with preoperative PSA-values and age at surgery.

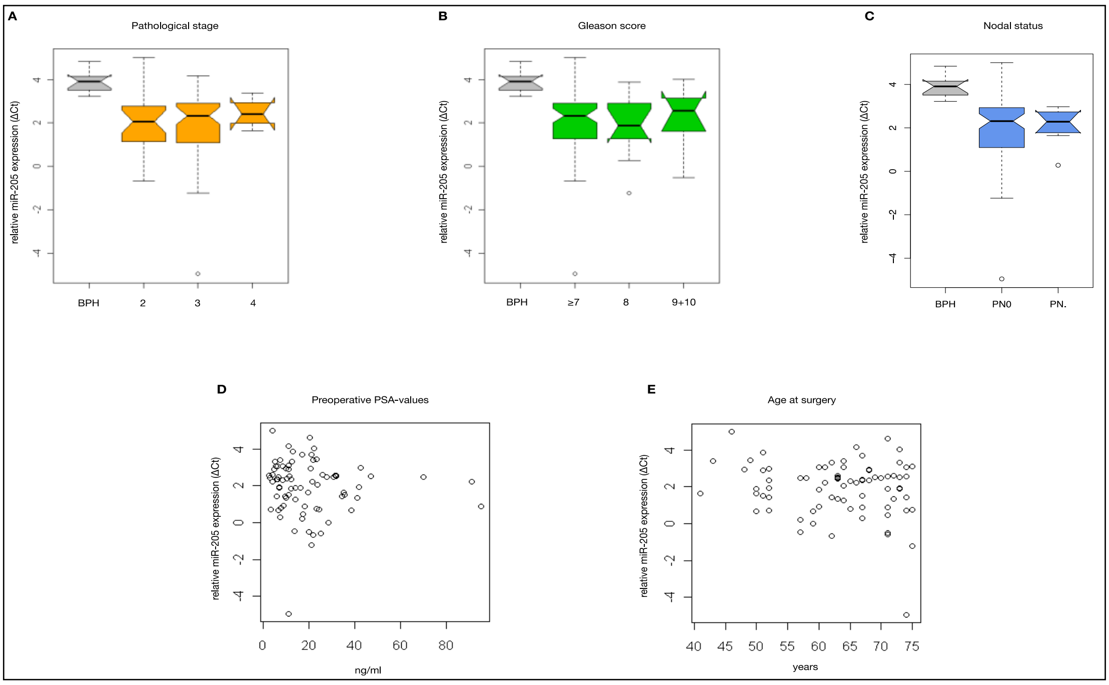

Figure S4. (**A**) Table indicating the 10-year survival estimation for overall survival (OS), cancer-specific survival (CSS), clinical failure free survival (CFFS) and biochemical recurrence-free survival (BCRFS) for the entire HRPCa study cohort (*n* = 105) and the HRPCa validation cohort (*n* = 78); (**B**) Kaplan-Meier estimates with 95% CI for overall survival (OS), cancer-specific survival (CSS), clinical failure free survival (CFFS) and biochemical recurrence-free survival (BCRFS) for the entire HRPCa study cohort **(left plots**, *n* = 105) and the HRPCa validation cohort (**right plots**, *n* = 78).

A

	OS (%)		CSS (%)		CFFS (%)		BCRFS (%)	
	study cohort	validation cohort	study cohort	validation cohort	study cohort	validation cohort	study cohort	validation cohort
10-years	61. 3	71. 1	87. 3	81. 5	66. 2	75. 6	41. 0	32. 2

B

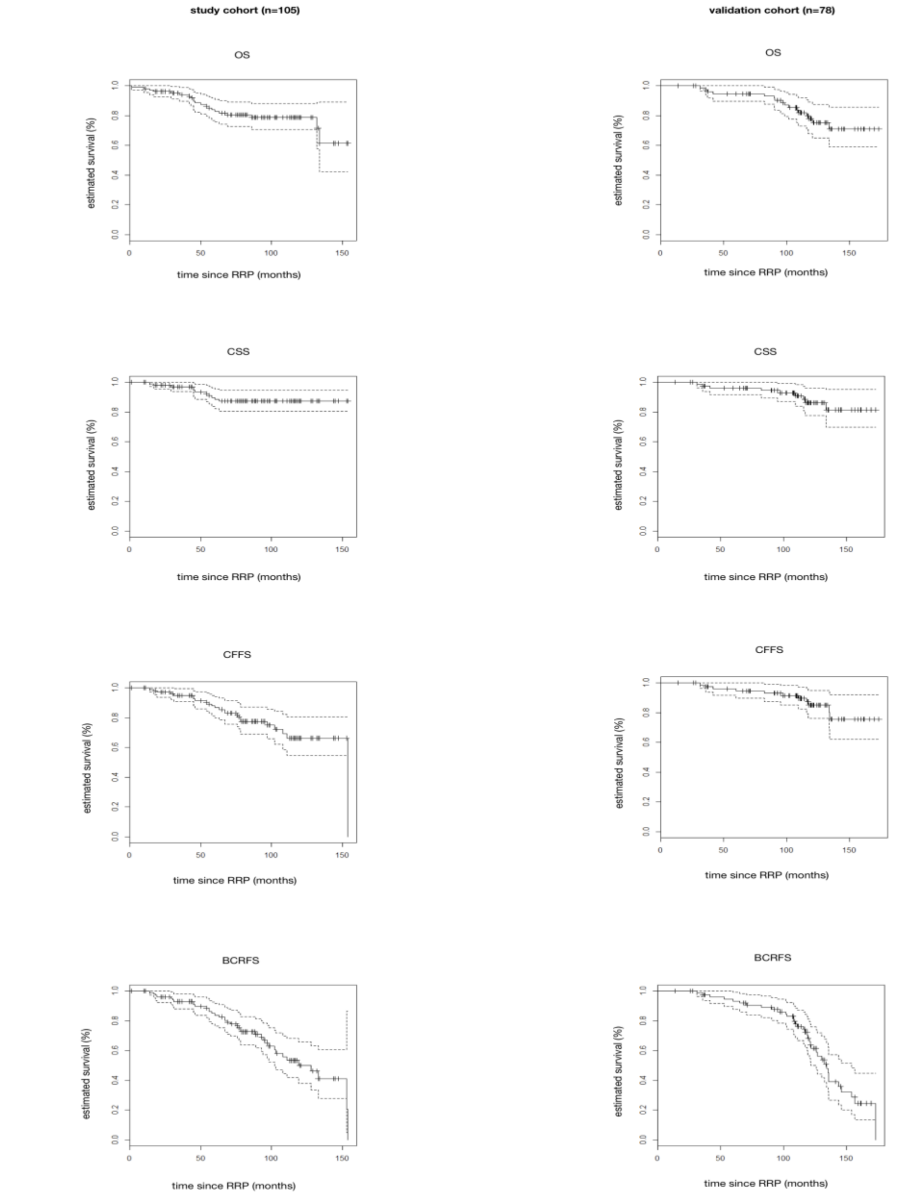

© 2013 by the authors; licensee MDPI, Basel, Switzerland. This article is an open access article distributed under the terms and conditions of the Creative Commons Attribution license (http://creativecommons.org/licenses/by/3.0/).
